# COVID-19, Sistema Renina-Angiotensina, Enzima Conversora da Angiotensina 2 e Nicotina: Qual a Inter-Relação?

**DOI:** 10.36660/abc.20200653

**Published:** 2020-10-13

**Authors:** Jaqueline Ribeiro Scholz, Marcelo Antônio Cartaxo Queiroga Lopes, José Francisco Kerr Saraiva, Fernanda Consolim Colombo

**Affiliations:** 1 Universidade de São Paulo Faculdade de Medicina Instituto do Coração do Hospital das Clinicas São PauloSP Brasil Instituto do Coração do Hospital das Clinicas da Faculdade de Medicina da Universidade de São Paulo, São Paulo, SP - Brasil; 2 Hospital Alberto Urquiza Wanderley Hemodinâmica e Cardiologia Intervencionista João PessoaPB Brasil Hospital Alberto Urquiza Wanderley - Hemodinâmica e Cardiologia Intervencionista, João Pessoa, PB - Brasil; 3 Hospital Metropolitano Dom José Maria Pires João PessoaPB Brasil Hospital Metropolitano Dom José Maria Pires, João Pessoa, PB - Brasil; 4 Sociedade Brasileira de Cardiologia Rio de JaneiroRJ Brasil Sociedade Brasileira de Cardiologia, Rio de Janeiro, RJ - Brasil; 5 Pontifícia Universidade Católica de Campinas CampinasSP Brasil Pontifícia Universidade Católica de Campinas, 5 Campinas, SP - Brasil

**Keywords:** COVID-19, Coronavirus/complicações, Betacoronavirus, SARS-CoV2, Syndrome Respiratory Acute, SARS-CoV2

A Organização Mundial da Saúde (OMS) declarou a COVID-19, infecção causada pelo novo Coronavírus (SARS-CoV-2),^1^ como uma pandemia em 11 de março de 2020. Até o início do mês de junho, foram contabilizados 7 milhões de casos positivos e cerca de 400 mil mortes pela doença no mundo.^2^ No Brasil, nesse mesmo período, foram aproximadamente 700 mil casos e cerca de 40 mil óbitos.^3^

Embora o vírus possa infectar indivíduos de qualquer idade, até o momento, a maioria dos casos graves foi descrita naqueles com mais de 55 anos, com comorbidades associadas, muitas delas cardiovasculares.^4^^,^^5^ Portanto, justifica-se a grande preocupação da comunidade médica em saber como atuar frente à COVID-19, em especial nessa população de maior risco e com muitas comorbidades cardiovasculares, com o objetivo de reduzir as taxas de morbimortalidade.^4^^,^^5^

O SARS-CoV-2 usa como receptor de entrada na célula a enzima conversora de angiotensina tipo 2 (ECA-2),^6^ uma molécula expressa em abundância na superfície das células do endotélio, dos rins, dos pulmões e de outros órgãos. Ela é um componente do sistema renina-angiotensina (SRA), cuja sequência genômica foi descoberta em 2000.^6^ A partir de então, foi possível reconhecer um eixo compensatório das ações clássicas do SRA (eixo “protetor”) para contrapor o eixo deletério causado pela produção da angiotensina 2. Do ponto de vista estrutural, a ECA-2 é semelhante à clássica; porém, do ponto de vista funcional, elas se contrapõem.^7^ Isso porque a ECA converte a angiotensina 1 em angiotensina 2 e provoca efeitos deletérios decorrentes da estimulação dos receptores AT1, como aumento da atividade simpática, reabsorção de sal e água, vasoconstrição, inflamação, liberação de aldosterona e vasopressina, contribuindo para fibrose tecidual, disfunção do endotélio e hipertensão arterial. A ECA-2 decompõe a angiotensina 2 em seus metabólitos, incluindo angiotensina (1 a 9) e angiotensina (1 a 7), e ativa receptores mas, que são potentes vasodilatadores e, portanto, podem ser um regulador negativo do SRA.^7^ A ECA-2 é expressa em uma variedade de tecidos diferentes, incluindo as vias respiratórias superiores e inferiores, o miocárdio e a mucosa gastrointestinal.^8^ Embora sua função na saúde e na doença humana não tenha sido totalmente elucidado, ela parece ter um importante papel regulador na pressão sanguínea e na função cardíaca. O papel fisiológico da ECA-2 nas vias respiratórias é ainda desconhecido; no entanto, em camundongos, foi demonstrado que ela protege de lesões pulmonares graves relacionadas a aspiração e sepse.^9^

As questões envolvendo a relação entre maior disponibilidade de receptores de ECA-2 e, possivelmente, maior susceptibilidade para a infecção pelo SARS-Cov-2^2^ são amplamente debatidas na cardiologia, visto que o uso de medicamentos como inibidores da enzima conversora de angiotensina (IECA) e bloqueadores do receptor de angiotensina 2 (BRA) aumenta a expressão dos receptores da ECA-2 em diferentes tecidos, incluindo o pulmão,^10^ embora seja fundamental para o tratamento da hipertensão arterial e insuficiência cardíaca.^11^^,^^12^ Houve discussões a respeito da substituição desses fármacos na vigência da pandemia; porém, devido à relevância no quesito eficácia e segurança no tratamento das doenças cardiovasculares e, até o momento, à ausência de evidências da relação entre o uso deles e o aumento da mortalidade pela COVID-19, há um consenso^13^ quanto à manutenção dos mesmos até haver evidências robustas que possam indicar o contrário. Na verdade, a boa notícia é que os estudos sugerem até mesmo um efeito protetor dos IECA na redução de mortalidade durante infecção pelo SARS-CoV-2, e nenhuma comprovação de aumento de risco em usuários de BRA.^14^

É interessante observar que outro aspecto muito relevante e polêmico também envolve a expressão da ECA-2 e se relaciona ao tabagismo. Alguns autores^15^^,^^16^ levantaram a hipótese de que a observação da baixa prevalência de fumantes internados com COVID-19 na China e na França, em comparação com a prevalência mais elevada do tabagismo na população geral, possa ter relação com a menor expressão de ECA-2, provocada pela nicotina.^17^ Oakes et al.^17^ fazendo uma revisão sobre efeitos da nicotina e SRA, demostrou que a nicotina inalada altera a homeostase do SRA pulmonar, por estimular seu eixo clássico (aumento de expressão e concentração de ECA-ANG 2) em detrimento do eixo protetor (redução de expressão e concentração de ECA-2 e angiotensina 1-7), determinando, assim, menor expressão de ECA-2. Desse modo, os defensores da hipótese^16^ do efeito “protetivo” da nicotina especulam que isso dificultaria a adesão do SARS-Cov-2 no epitélio respiratório. Cabe ressaltar que a média de idade dos pacientes internados com COVID-19 é mais elevada,^4^ e a prevalência do tabagismo cai significativamente com o envelhecimento, tanto porque fumantes morrem precocemente^18^ quanto porque deixam de fumar quando adoecem.^18^ Novamente, esse é um paradoxo envolvendo a expressão dos receptores de ECA-2 e o SRA.

Nesse contexto, algumas perguntas devem ser respondidas: existem dados epidemiológicos que indiquem esse efeito “protetor”? Qual é a ação da nicotina sobre o SRA no epitélio brônquico? A relação entre expressão de ECA-2 no epitélio pulmonar é semelhante entre fumantes e não fumantes? Quais as consequências da interrupção na homeostase do SRA no pulmão causada pela nicotina?

Os dados de mortalidade mostram maior risco de morte por COVID-19 em fumantes com ou sem doença pulmonar obstrutiva crônica (DPOC),^19^^-^^21^ e o risco de intubação é dobrado^19^ comparando-se fumantes com não fumantes. Esses dados corroboram o que ocorre em outras infecções virais, cujo curso é pior em fumantes.^22^^,^^23^ Considerando-se a complexidade do SRA, a nicotina pode afetar outros elementos além dos discutidos, provocando efeitos ainda não elucidados.

Estudos recentes demonstraram aumento da expressão da ECA-2 no epitélio de pequenas vias respiratórias de tabagistas e portadores de DPOC com COVID-19. Brake et al.,^24^ por meio da imuno-histoquímica, identificaram pela primeira vez uma expressão aumentada de ECA-2 no tecido pulmonar de pacientes com COVID-19. Porém ela foi maior nos pacientes com DPOC, fumantes ou não, e em menor proporção em fumantes sem DPOC. Não se encontrou aumento da expressão de ECA-2 em indivíduos não fumantes. Leung et al.^25^ também observaram maior expressão da ECA-2 no epitélio das pequenas vias respiratórias de pacientes com DPOC e fumantes com COVID-19, analisando material de lavado brônquico e correlacionando isso à gravidade da doença. Russo et al.^26^ investigaram *in vitro* o mecanismo pelo qual a nicotina poderia levar ao aumento da ECA-2 nessa população. Diferentes células das vias respiratórias, como epiteliais brônquicas, epiteliais alveolares tipo 2 e fibroblastos intersticiais, expressam receptores nicotínicos de acetilcolina (nAChR), especificamente o subtipo α7-nAChR, e também os componentes do SRA. Quantificando-se a expressão de ECA-2 em células epiteliais brônquicas em cultura, foi possível demonstrar que a nicotina promove regulação positiva (aumento da expressão de ECA-2) mediada especificamente pela sua ligação com os receptores α7-nAChR. Assim, o tabagismo poderia provocar um aumento no mecanismo de captação celular para o SARS-Cov-2 por meio da sinalização da via α7-nAChR.

**Figura 1:**
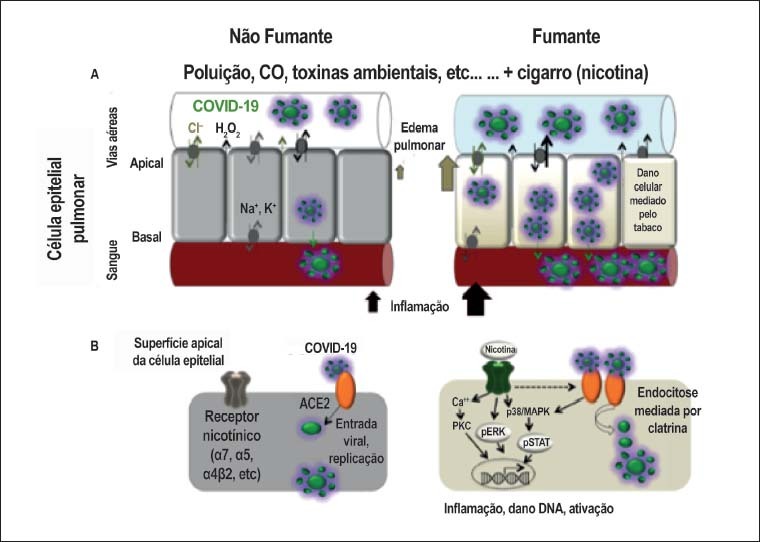
Modelo esquemático de como a exposição à nicotina aumenta o risco de entrada do SARS-CoV-2 no pulmão do hospedeiro humano. A. Respostas pulmonares e imunes à infecção pelo vírus em células epiteliais de fumantes (à direita) e não fumantes (à esquerda). B. Mecanismos celulares desencadeados pela atividade de receptores nicotínicos promovem a entrada e proliferação do SARS-CoV-2 nas células epiteliais por meio da coexpressão da ECA-2. A ativação de receptores nicotínicos pela nicotina pode causar maior ativação de proteases, morte celular (apoptose) e sinalização inflamatória por meio de mecanismos que convergem nas vias de regulação e sinalização da ECA-2.

Com esses dados, o raciocínio seria de que os pacientes tabagistas e com DPOC teriam, na verdade, mais susceptibilidade à infecção pelo SARS-Cov-2. Esse mecanismo, inclusive, foi formulado e representado em modelo esquemático (Figura 1) por Olds e Kabbani^27^ e explica como a exposição à nicotina aumenta o risco de entrada do vírus nas células pulmonares e, consequentemente, como o ato de fumar pode ter impacto negativo na fisiopatologia da COVID-19.

Nesse contexto, pode-se interpretar que o papel do SRA na gravidade da infeção por SARS-CoV-2 depende menos da expressão da ECA-2 no aparelho cardiovascular e mais da expressão dele no epitélio respiratório. Isso pode justificar a não interferência na morbimortalidade por COVID-19 em usuários de IECA e BRA, bem como a falta de proteção para essa doença em tabagistas e pacientes com DPOC.

Além de as orientações terapêuticas serem parar de fumar e manter a medicação cardiovascular, o conhecimento da inter-relação da nicotina com a expressão de ECA-2 nas células do epitélio respiratório e sua interface com receptores de α7-nAChR sugere a possibilidade de ações terapêuticas para tratamento da COVID 19. O uso de antagonistas seletivos de α7-nAChR, como metilglicaconitina^28^ e α-conotoxina,^29^ pode alterar significativamente a expressão de ECA-2, podendo ser uma opção terapêutica para impedir a entrada de SARS-CoV-2 no epitélio das vias respiratórias. Futuros estudos deverão confirmar ou não essas hipóteses.
