# Supplementary material for: COVID-19, Sistema Renina-Angiotensina, Enzima Conversora da Angiotensina 2 e Nicotina: Qual a Inter-Relação?
Source: Arq Bras Cardiol. 2020 Oct 13;115(4):708–11. [Article in Portuguese] doi: 10.36660/abc.20200653 (PMC8386966; doi:10.36660/abc.20200653)
Supplement: Translation

# Translation: COVID-19, Renin-Angiotensin System, Angiotensin-Converting Enzyme 2, and Nicotine: What is the Interrelation?

**Authors:** Jaqueline Ribeiro Scholz, Marcelo Antônio Cartaxo Queiroga Lopes, José Francisco Kerr Saraiva, Fernanda Consolim Colombo

**Affiliations:** 1 São PauloSP Brazil Instituto do Coração do Hospital das Clinicas da Faculdade de Medicina da Universidade de São Paulo, São Paulo, SP - Brazil; 2 João PessoaPB Brazil Hospital Alberto Urquiza Wanderley - Hemodinâmica e Cardiologia Intervencionista, João Pessoa, PB - Brazil; 3 João PessoaPB Brazil Hospital Metropolitano Dom José Maria Pires, João Pessoa, PB - Brazil; 4 Rio de JaneiroRJ Brazil Sociedade Brasileira de Cardiologia, Rio de Janeiro, RJ - Brazil; 5 CampinasSP Brazil Pontifícia Universidade Católica de Campinas, Campinas, SP - Brazil

**Keywords:** COVID-19, Coronavirus/complicações, Betacoronavirus, SARS-CoV2, Syndrome Respiratory Acute, SARS-CoV2

The World Health Organization (WHO) declared COVID-19, an infection caused by the new Coronavirus (SARS-CoV-2),^1^ as a pandemic on March 11, 2020. By the beginning of June, 7 million positive cases and around 400 thousand deaths from the disease had been reported worldwide.^2^ In the same period, Brazil accounted for approximately 700 thousand cases and 40 thousand deaths.^3^

Although the virus can infect individuals of any age, so far the most serious cases have been described in people aged 55 and over, with associated comorbidities—many of them related to the cardiovascular system.^4^^,^^5^ Therefore, the medical community’s great concern about knowing how to act against COVID-19 is justifiable, especially in this population at higher risk and with many cardiovascular comorbidities, and the aim is reducing morbidity and mortality rates.^4^^,^^5^

SARS-CoV-2 uses as a cell receptor the angiotensin-converting enzyme type 2 (ACE-2),^6^ a molecule abundantly expressed on the surface of cells in the endothelium, kidneys, lungs, and other organs. It is a component of the renin-angiotensin system (RAS), whose genomic sequence was discovered in 2000.^6^ Since then, a compensatory axis of the classic actions of the RAS (“protective” axis) was recognized to counteract the harmful axis caused by production angiotensin 2. From a structural point of view, ACE-2 is similar to the classic one; but, from the functional point of view, they are opposed.^7^ This is because ACE converts angiotensin 1 into angiotensin 2 and causes deleterious effects resulting from the stimulation of AT1 receptors, such as increased sympathetic activity, salt and water reabsorption, vasoconstriction, inflammation, aldosterone and vasopressin release, all contributing to tissue fibrosis, endothelial dysfunction, and arterial hypertension.

ACE-2 breaks down angiotensin 2 into its metabolites, including angiotensin (1 to 9) and angiotensin (1 to 7), and activates MAS receptors (MasR), which are potent vasodilators and therefore can be a negative regulator of SARS.^7^ ACE-2 is expressed in a variety of different tissues, including the upper and lower airways, the myocardium and the gastrointestinal mucosa.^8^ Although its function in human health and disease has not been fully elucidated, it appears to have an important regulatory role in blood pressure and cardiac function. The physiological role of ACE-2 in the airways is still unclear, but, in mice, it was shown to protect against severe lung injury related to aspiration and sepsis.^9^

The issues underlying the relationship between increased availability of ACE-2 receptors and possibly greater susceptibility to SARS-Cov-2^2^ infection are widely debated in cardiology, as the use of drugs as angiotensin-converting enzyme (ACE) inhibitors and angiotensin 2 receptor blockers (ARB) increases the expression of ACE-2 receptors in different tissues such as the lung,^10^ although it is fundamental for the treatment of arterial hypertension and heart failure.^11^^,^^12^ Discussions about the replacement of these drugs have taken place during the pandemic; however, due to their relevance in terms of efficacy and safety in the treatment of cardiovascular diseases and, to date, the absence of evidence of a relationship between their use and the increase in mortality by COVID-19, there is a consensus^13^ regarding their maintenance until there is reliable evidence that indicate otherwise. In fact, the good news is that studies even suggest a protective effect of ACE inhibitors in reducing mortality during SARS-CoV-2 infection, and no evidence of increased risk in ARB users.^14^

It is interesting to note that another very relevant and controversial aspect also involves the expression of ACE-2 and is related to smoking. Some authors^15^^,^^16^ have raised the hypothesis that the low prevalence of smokers hospitalized with COVID-19 in China and France, in comparison with the higher prevalence of smoking in the general population, may be related to the lower expression of ACE-2 caused by nicotine.^17^ Oakes et al.^17^ reviewing the effects of nicotine and RAS, demonstrated that inhaled nicotine alters the SARS pulmonary homeostasis by stimulating its classic axis (increased expression and concentration of ACE-2) to the detriment of the protective axis (reduced expression and concentration of ACE-2 and angiotensin 1-7), thus determining less expression of ACE-2. Thus, supporters of the hypothesis^16^ of a “protective” effect of nicotine speculate that this would make it difficult for SARS-Cov-2 to adhere to the respiratory epithelium. It should be noted that the average age of patients hospitalized with COVID-19 is higher,^4^ and the prevalence of smoking drops significantly with aging, because smokers either die early^18^ or stop smoking when they get sick.^18^ Again, this is a paradox involving the expression of ACE-2 receptors and RAS.

In this context, some questions remain unanswered: are there epidemiological data that indicate this “protective” effect? What is the action of nicotine on the RAS in the bronchial epithelium? Is the relationship between ACE-2 expression in the pulmonary epithelium similar between smokers and non-smokers? What are the consequences of the interruption in RAS homeostasis by nicotine in the lung?

Data on mortality show a higher risk of death from COVID-19 among smokers with or without chronic obstructive pulmonary disease (COPD),^19^^-^^21^ and the risk of intubation is doubled^19^ when comparing smokers with non-smokers. These data corroborate what occurs in other viral infections, with a worse course in smokers.^22^^,^^23^ Considering the complexity of RAS, nicotine can affect elements other than those discussed, causing effects not yet elucidated.

Recent studies have shown increased expression of ECA-2 in the epithelium of small airways in smokers and COPD patients with COVID-19. Brake et al.,^24^ using immunohistochemistry, identified for the first time an increased expression of ACE-2 in the lung tissue of patients with COVID-19. However, it was higher in COPD patients, whether they were smokers or not, and was present in a lesser extent in smokers without COPD. There was no increase in the expression of ACE-2 in nonsmokers. Leung et al.^25^ also reported a greater expression of ACE-2 in the epithelium of the small airways of patients with COPD and smokers with COVID-19, analyzing bronchial lavage material and correlating this with the severity of the disease. Russo et al.^26^ investigated *in vitro* the mechanism by which nicotine could lead to an increase in ACE-2 in this population. Different airway cells, such as bronchial epithelial cells, type 2 alveolar epithelial cells, and interstitial fibroblasts express nicotinic acetylcholine receptors (nAChR), specifically the α7-nAChR subtype, and also the components of the RAS. By quantifying the expression of ECA-2 in cultured bronchial epithelial cells, they demonstrated that nicotine promotes a positive regulation (increased expression of ACE-2) mediated specifically by its binding with α7-nAChR receptors. Thus, smoking could cause an increase in the cellular uptake mechanism for SARS-Cov-2 by signaling the α7-nAChR pathway.

With these data, the reasoning would be that patients who smoke and have COPD would, in fact, be more susceptible to SARS-Cov-2 infection. This mechanism was formulated and represented in a schematic model (Figure 1) by Olds and Kabbani^27^ and explains how exposure to nicotine increases the risk of the virus entering the lung cells and, consequently, how smoking can have a negative impact in the pathophysiology of COVID-19.

**Figure 1:** Schematic model of how exposure to nicotine increases the risk of SARS-CoV-2 entering the lung of the human host. A. Pulmonary and immune responses to virus infection in smokers’ (right) and nonsmokers’ (left) epithelial cells. B. Cellular mechanisms triggered by the activity of nicotinic receptors promote the entry and proliferation of SARS-CoV-2 in epithelial cells through the co-expression of ACE-2. The activation of nicotinic receptors by nicotine can cause greater activation of proteases, cell death (apoptosis) and inflammatory signaling through mechanisms that converge in ACE-2 regulation and pathways signaling.

In this context, we can interpret that the role of the RAS in the severity of SARS-CoV-2 infection depends less on the expression of ACE-2 in the cardiovascular system and more on its expression in the respiratory epithelium. This may justify the non-interference in COVID-19 morbidity and mortality in ACEI and ARB users, as well as the lack of protection for this disease in smokers and COPD patients.

In addition to the therapeutic guidelines being to quit smoking and maintain cardiovascular medication, knowledge about the interrelation of nicotine with the expression of ACE-2 in the cells of the respiratory epithelium and its interface with α7-nAChR receptors suggests the possibility of therapeutic actions for the treatment of COVID 19. The use of selective α7-nAChR antagonists such as methylglycaconitin^28^ and α-conotoxin^29^ can significantly alter the expression of ACE-2, and may be a therapeutic option to prevent the entry of SARS-CoV-2 in the respiratory tract epithelium. Further studies should confirm or not these hypotheses.
